# Effect of Moisture and Oil Content in the Supercritical CO_2_ Defatting of *Hermetia illucens* Larvae

**DOI:** 10.3390/foods12030490

**Published:** 2023-01-20

**Authors:** Tiziana Fornari, Luis Vázquez, David Villanueva-Bermejo, Raúl Hurtado-Ribeira, Diego Martín Hernández, Diana Martin

**Affiliations:** 1 Institute of Food Science Research CIAL (CSIC-UAM), Sección Departamental de Ciencias de la Alimentación, Facultad de Ciencias, UAM, CEI UAM+CSIC, C/Nicolás Cabrera 9, Campus de Cantoblanco, 28049 Madrid, Spain; 2 Department of Production and Characterization of Novel Foods, Institute of Food Science Research (CIAL, CSIC-UAM), C/Nicolas Cabrera 9, Cantoblanco Campus, Autonomous University of Madrid, 28049 Madrid, Spain

**Keywords:** *H. illucens*, edible insects, oil, defatting, supercritical fluid extraction

## Abstract

The supercritical defatting of *H. illucens* was scaled up at 450 bar and 60 °C from a 270 cm^3^ extraction cell to a vessel five times larger. Then, eight different *H. illucens* larvae batches, with variable content of oil (16.80–29.17% w/w) and moisture (4.45–15.95% w/w) were defatted. The effect of these parameters on yield and oil composition was analyzed. The presence of moisture in the larvae batch, in the range of the values studied, had no negative effect on the oil recovery efficiency, which was mainly determined by the initial content of oil in the larvae samples. Furthermore, no differences were determined in the fatty acid profile of the oils recovered, which were rich in saturated fatty acids, mainly lauric acid (ca. 50% w/w). Minor lipids, such as squalene and phytosterols, were determined in all the oil samples. The moisture content in the oils extracted was in the range of 0.118–1.706% w/w. Therefore, some samples exceeded the limits recommended for volatile matter in edible fats and oils (0.2%, including moisture). Yet, concerning the oil peroxide index, values were much lower than those corresponding to the oil extracted using hexane.

## 1. Introduction

The world population is forecasted to be 10 billion in 2050; thus, a lack of animal protein to supply the future world demand is expected. Edible insects are currently alternative food sources, with adequate nutritional requirements, not only regarding proteins, but also lipids and other micronutrients [1]. 

The European Food Safety Authority (EFSA) considers whole insects and their parts as novel foods since January 2018, according to the new EU regulation [2]. Currently, four insect species (*Tenebrio molitor*, *Locusta migratoria, Acheta domesticus*, and *Alphitobius diaperinus*) have already been approved for human diet since 2021 [3,4,5], and more species are expected to be authorized in the comin months, such as *H. illucens*, *Gryllodes sigillatus,* or *Apis mellifera*. Additionally, most of these insect species are already authorized for fish, poultry, and pig feeding.

Nevertheless, cultural and psychological barriers may affect consumer acceptance of insects as food, particularly in the European member states. Therefore, the incorporation of edible insect powders into familiar food products is proposed as a solution to increase acceptance and consumption of edible insects [6]. Another issue arises from the usual high lipid content of some insect species, which may interfere with the production of protein-rich products. Therefore, a lipid separation step is often required. In the case of animal feeding, the defatting process is also necessary for insect species with a high lipid content, since fat may hinder the processing of the feeds, affect the palatability, and impact the productive yields and lipid composition of the fed animals.

Black soldier fly *(H. illucens)* larvae are among the popular edible insects currently farmed and studied for food and feed in Europe [7,8,9]. Moreover, bioactive properties of *H. illucens* have been recently investigated and reported [10,11,12]. *H. illucens* larvae contain around 40% w/w of proteins rich in essential amino acids, and more than 28% w/w of lipid compounds, together with relevant amounts of minerals, such as calcium and phosphorous [7]. As coconut oil, *H. illucens* oil presents a high medium-chain fatty acid content, particularly lauric acid, which is well known for its antimicrobial effect on intestinal bacteria [13,14]. Therefore, an appropriate defatting process of *H. illucens* larvae would produce the expected protein-rich products, as well as the insect oil, with high commercial opportunity exploitation. Furthermore, it has been generally assumed that a previous defatting process is necessary for the production of insect flours with adequate bioactivities, such as antioxidant or antimicrobial activities [12,15,16]. 

Since the emerging insect industry is growing according to sustainability principles [17,18], environmentally clean processes for larvae defatting are also demanded. In this respect, supercritical fluid extraction (SFE) using carbon dioxide (CO_2_) is a well-known technology for the defatting of vegetal materials [19] due to the high selectivity of supercritical CO_2_ to extract lipid compounds. In addition, CO_2_ is completely removed after the extraction, allowing the production of solvent-free oils, minimizing oil post-treatment. Therefore, supercritical CO_2_ extraction could also be an efficient and environmentally clean technique for the defatting of insects. However, the use of SFE for the defatting of edible insects has been scarcely studied. Nevertheless, some examples have been reported in the last years, showing the potential of this technology. Kim et al. [20] studied the fat removal from *H. illucens* to enable the larvae to be used as a feedstock, e.g., for aquaculture and livestock. The use of SFE on crushed larvae reduced the fat content to ca. 5% w/w at 350 bar and 35 °C. The studies conducted by Kim et al. [20], Cantero-Bahillo et al. [21], and Laurent et al. [22] are currently the only ones that have provided information on the defatting of *H. illucens* larvae using supercritical CO_2_. In addition, Laroche et al. [15] investigated different defatting methods of house cricket (*A. domesticus*) and mealworm (*T. molitor*) flours, concluding that supercritical CO_2_ allowed obtaining similar lipid extraction yields to those obtained using conventional methods (solid–liquid extraction with hexane, petroleum ether, ethyl acetate, and ethanol) from *T. molitor.* However, it was less efficient in the case of *A. domesticus*. Purschke et al. [23] investigated the SFE defatting of *T. molitor* larvae, studying the influence of extraction conditions in a pilot-scale unit, and demonstrating that the defatting performance and oil composition were not substantially different when hexane was used as solvent in comparison to supercritical CO_2_. In a study recently reported by our research group [21], the kinetic behavior in the SFE extraction of fat from *H. illucens* larvae at different pressures and temperatures was investigated. Dry larvae (moisture content close to 5% w/w) containing 47.3% and 34.5% w/w of oil were satisfactorily defatted with supercritical CO_2_. The highest oil recovery (95% and 98%, respectively) was achieved at 450 bar and 60 °C. Furthermore, it was concluded that the composition of the oil extracted was not significantly different from the one obtained with hexane. 

It is well known that the moisture content in the raw material has a great impact on the supercritical extraction of oil [24]. The presence of water might be positive due to the expansion of the solid matrix and, thus, the increased permeability, or it might be negative as a result of the hindrance to the oil diffusion through the matrix. Taking into account the emerging use of SFE in the defatting of insects, especially for *H. illucens*, there is a need for further investigations of this procedure to understand how the composition of the matrix, in terms of oil and moisture content, could modulate the efficacy of the defatting and the quality of the oil obtained. In the present study, the optimal process conditions established previously [21] were applied to scale up the defatting by SFE of different batches of *H. illucens* larvae with variable content of oil (16.80–29.17% w/w) and moisture (4.45–15.95% w/w), analyzing the effect of these parameters on oil recovery and composition. 

## 2. Materials and Methods

### 2.1. Samples and Reagents

Eight *H. illucens* batches with 16.80–29.17% w/w oil and 4.45–15.95% w/w moisture content were kindly supplied by Entomo Agroindustrial S.A. (Cehegin, Spain) to study the effect of moisture and oil content in the supercritical defatting process. Prior to the extraction, the insect larvae were ground in a knife mill (Grindomix GM 200, Retsch GmbH, Haan, Germany). 

Absolute ethanol was purchased from Panreac (Barcelona, Spain). Methanol, hexane (95%), and ethyl acetate were purchased from Macron (Gliwice, Poland). N,O-bis-(trimethylsilyl)trifluoro- acetamide (BSTFA) was purchased from Sigma-Aldrich Chemie GmbH (Steinheim, Germany). CO_2_ (N-38) was obtained from Carburos Metalicos, S.A. (Madrid, Spain).

### 2.2. Determination of Moisture Content

Determination of moisture content in larvae: Moisture content was gravimetrically determined by heating 2 g of sample in an oven, drying at 105 °C until constant weight. The weight loss was recorded to calculate the initial moisture content of *H. illucens* flours. The experimental assays were carried out in duplicate.

Determination of moisture content in the extracted oil: Moisture content was determined according to the ISO 662 method [25]. A weight of 4 g of sample was placed in a capsule and dried in an oven at 103 °C for 1 h. Then, the samples were cooled to room temperature in a desiccator, and the weight loss was determined. The process was repeated in successive 30 min heating time intervals until the mass of sample was constant. The experimental assays were carried out in duplicate.

### 2.3. Determination of Fat Content by Hexane Solvent Extraction

A conventional solid–liquid extraction was performed with 95% hexane in a homogenizer (Ultra-turrax T18 basic, IKA, Staufen, Germany) at 11,000 rpm for 5 min. A ground larva-to-solvent ratio of 1:5 (w/v) was applied. Then, the mixture was centrifuged at 3400× *g* for 10 min and 20 °C. The supernatant was collected, and the precipitate was defatted again following the same procedure. Hexane was removed using a vacuum rotary evaporator. Fat yield (%) was calculated as the ratio of the weight of extracted fat with respect to the weight of initial sample. Defatting using hexane was performed in duplicate.

### 2.4. Defatting of H. illucens Flour by Supercritical CO_2_

#### 2.4.1. Kinetic Study and Process Scale Up

**Figure 1:**
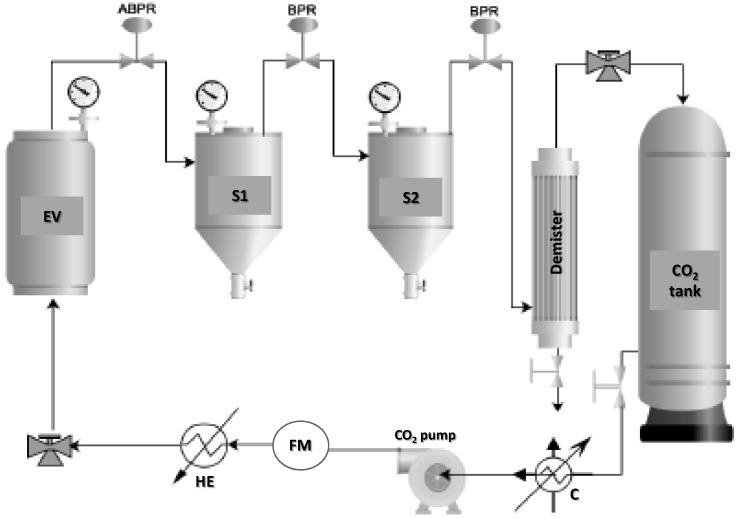
Schematic diagram of the SFE plant: EV, extraction vessel; S1, first separator; S2, second separator; ABPR, automatic back pressure regulator; BPR, back pressure regulator; C, CO_2_ cooler; FM, flow meter; HE, heat exchanger.

The extraction kinetics was studied in a pilot-scale supercritical fluid extractor (Thar Technology, Pittsburgh, PA, USA, model SF2000) comprising a 1350 cm^3^ cylinder extraction vessel (internal diameter of 0.07 m; height of 0.388 m) and two different separators (S1 and S2), each of 500 cm^3^ capacity, with independent control of temperature (±2 K) and pressure (±0.1 MPa). The maximum operating pressure and temperature in the extraction vessel were, respectively, 700 bar and 80 °C. The extraction unit also included a recirculation system, where CO_2_ was condensed and pumped up to the desired extraction pressure. The pressure in the extraction vessel was controlled by an automated back pressure regulator valve. The CO_2_ flow was measured using a flow meter from Siemens AIS (Model: Sitrans F C Mass 2100 DI 1.5, Nordborgvej, Denmark). A schematic diagram of the experimental device is given in Figure 1; a detailed description of the experimental device and its PLC-based instrumentation was provided in previous work [26]. 

Decompression of the supercritical outlet stream to the recirculation pressure (45 bar) was accomplished in the separators at 40 °C. In general, the mass of material recovered in S2 was less than 5% of the mass recovered in S1. 

Following previous studies [21], the kinetic data were obtained at 450 bar and 60 °C. CO_2_ flow rates were set to 85 and 130 g/min. The material extracted at the different time intervals was collected from S1 and the yield was calculated according to the following equation:

(1) $$Yield=\frac{mass\;of\;oil\;recovered\;in\;S1\;\left(g\right)}{mass\;of\;flour\;feed\;in\;the\;extraction\;vessel\;\left(g\right)}\times 100.$$

#### 2.4.2. Supercritical Extraction of Different *H. illucens* Larvae Batches 

The supercritical extraction of larvae batches with different initial oil and moisture content was carried out in the 1350 cm^3^ vessel. The extraction pressure and temperature were 450 bar and 60 °C for all the experiments. The extraction vessel was loaded with ca. 450 g of ground larvae. CO_2_ flow rate and extraction time were constant for all the experiments and selected according to the results obtained in the process scale-up study (Section 2.4.1). 

Decompression of the extract was accomplished as described before. Samples were collected and stored at −20 °C in the dark until analysis. The degree of defatting was calculated according to two different methods, as indicated in the following equations:

(2) $$D1=\frac{mass\;recovered\;in\;S1\;\left(g\right)}{mass\;of\;oil\;in\;larvae\;sample\;\left(g\right)}\times 100.$$

(3) $$D2=\frac{mass\;of\;oil\;in\;larvae\;sample\;\left(g\right)-mass\;of\;oil\;in\;defatted\;flour\;\left(g\right)}{mass\;of\;oil\;in\;larvae\;sample\;\left(g\right)}\times 100.$$

In Equation (2), it is assumed that all the material recovered in S1 is oil, while, in Equation (3), the degree of defatting is calculating taking into account the initial and final amount of oil in the insect flour, i.e., before and after the supercritical extraction, which were experimentally determined as described in Section 2.3 (hexane extraction).

### 2.5. Analysis of the Extracted Oil 

Fatty acid profile: The analysis of fatty acids, either free fatty acids or constituting triglycerides, was carried out by gas chromatography (GC), according to the method of Vázquez et al. [27]. In a previous step, fatty acids were transformed into their corresponding fatty acid methyl esters (FAMEs). The methylation of fatty acids was carried out according to the AOAC Official Method 996.01 (Section E), using NaOH/methanol solution (0.5 N) and BF_3_/methanol solution (~14%, *w/v*) as catalysts. The FAMEs were analyzed by GC according to the method described by Vázquez et al. [27]. Identification and quantification of FAMEs were carried out in an Agilent 6850 Network GC System (Avondale, AZ, USA), coupled to an FID detector and Agilent 6850 autosampler. The capillary column was an HP-88 (30 m, 0.25 mm i.d.) (Avondale, AZ, USA). An injection volume of 1 μL and a 20:1 split ratio were used. The injector and detector temperatures were 220 and 250 °C, respectively. The temperature program started at 50 °C, rising to 220 °C at 15 °C·min^−1^. The final temperature (220 °C) was held for 10 min. Identification of FAMEs was based on the retention times and the relative area percentages of No. 3 PUFA reference standard (47085-U), obtained from Supelco (Bellefonte, PA, USA). For quantification, a calibration curve with concentrations ranging from 0.2 to 8.0 mg/mL of methyl linoleate was used. The equation of the calibration curve was as follows: mg injected = 1.2 × 10^–6^ × area.

Minor lipid compounds: Extracts were characterized by GC–MS after derivatization of the samples with BSTFA according to Herrera et al. [28] with small modifications. Extracts were dissolved in BSTFA at a concentration of 10 mg/mL and heated at 75 °C for 1 h. Samples rested at room temperature for 10 min and then were analyzed in an Agilent 7890A GC–MS (Agilent Technologies, Santa Clara, CA, USA). The column employed was an Agilent HP-5MS UI capillary column (30 m × 0.250 mm × 0.25 μm), and the carrier gas was helium with a flow of 2 mL/min. A G4513A autoinjector was used, with 1 μL injections in splitless mode, and the injector temperature was 260 °C. The oven was initially set at 50 °C and increased at 10 °C/min to 310 °C, before being held for 25 min. The inlet temperatures at the MS were set at 260 °C, and those at the MS ion source and the interface were 230 °C and 280 °C, respectively. The scanning speed was 0.79 scans/s in a mass range of 30–1000 amu. Identification of compounds was performed by the NIST MS Data library, by the mass spectra according to literature or according to commercial standards, previously derivatized following the same procedure as samples.

Peroxide value: The oxidative state of samples was measured immediately after determination of the peroxide value using the photometric FoodLab instrument (CDR S.r.L., Ginestra Fiorentina) according to Martín et al. [29]. 

## 3. Results

### 3.1. Kinetic Study and Process Scale-Up

In a previous study [21], the defatting of *H. illucens* larvae with high content of oil (47.3% w/w oil) was studied in a small-scale (SS) 270 cm^3^ extraction vessel loaded with 100 g of ground larvae. The defatting was tested at two different pressures (350 and 450 bar), temperatures (50 and 60 °C), and CO_2_ mass flow rates (60 and 100 g/min). A degree of defatting of at least 90% was obtained at 450 bar and 60 °C with both CO_2_ flow rates assessed. These pressure and temperature conditions were applied in the large-scale (LS) 1350 cm^3^ unit, and a suitable CO_2_ flow rate was scaled up in order to reproduce the same extraction kinetic.

López-Padilla et al. [30,31] studied the CO_2_ flow rate required for scaling up different plant matrices with the same small-scale and large-scale units used in this work. The study was based on the two well-known semi-empirical engineering approaches generally postulated to maintain the same extraction kinetics in two different SFE units, i.e., maintaining the same CO_2_ linear velocity (*v*) or maintaining the CO_2_ residence time (*t_R_*) in the SFE packed beds. According to their results, a satisfactory scaling of the CO_2_ flow rate was attained using the *t_R_* criteria:

(4) $$Q_{LS}={\left(\frac{D_{LS}}{D_{SS}}\right)}^{2}{\left(\frac{L_{LS}}{L_{SS}}\right)}^{}Q_{SS},$$

where *D* and *L* are, respectively, the internal diameter and length of the extractor vessel, and *Q* is the CO_2_ flow rate in the small-scale (*SS*) and large-scale (*LS*) units. Table 1 shows the results of applying the *t_R_* criteria (Equation (4)) in the case of the *H. illucens* larvae defatting studied in this work. As it can be observed in Table 1, with 60 g/min in the SS unit, a CO_2_ flow rate of 270 g/min is required in the *LS* unit to maintain the same *t_R_* and, thus, the same extraction kinetics. Unfortunately, due to limitations in the available CO_2_ pump, this large CO_2_ flow rate could not be tested. Thus, the CO_2_ flow rates selected to carry out large-scale kinetic studies were 85 and 130 g/min, both suitable operating flow rates for the available CO_2_ pump.

**Figure 2:**
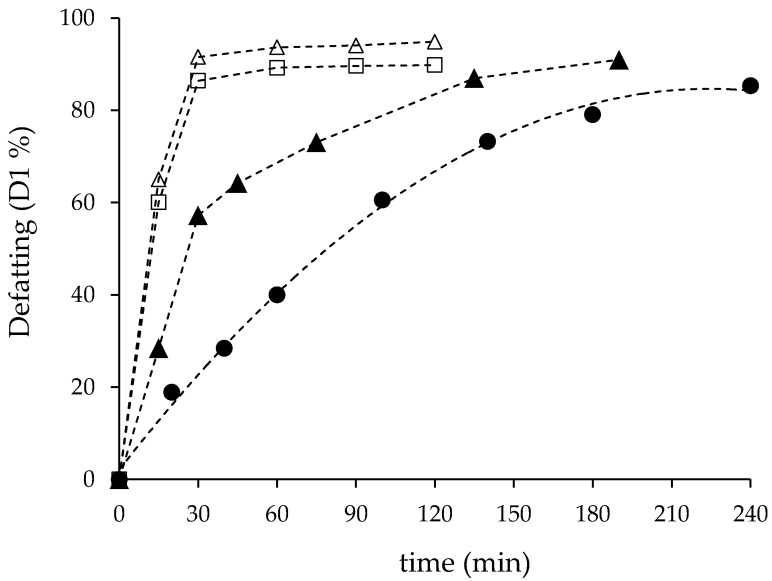
Comparison of the defatting kinetic of *H. illucens* larvae at 450 bar and 60 °C: (△) 270 cm^3^ vessel and 100 g/min CO_2_; (☐) 270 cm^3^ vessel and 60 g/min CO_2_; (⬤) 1350 cm^3^ vessel and 85 g/min CO_2_; (▲) 1350 cm^3^ vessel and 130 g/min CO_2_; (- - -) trend line.

Figure 2 provides a comparison of the kinetic data obtained at 450 bar and 60 °C using the 270 and 1350 cm^3^ vessels and different CO_2_ flow rates and CO_2_/larvae ratios (CO_2_/larvae = Q × extraction time/F). In the SS extractor, after 90 min, an oil recovery of 94% was obtained using 100 g/min of CO_2_ (CO_2_/larvae ratio = 90 g/g) and ca. 90% with 60 g/min (CO_2_/larvae ratio = 54 g/g). The fastest extraction velocity was attained in the SS unit, with a recovery higher than 85% just in 30 min. 

As expected, in the LS vessel, none of the two CO_2_ flow rates used (85 and 130 g/min) could reproduce the fast extraction attained in the SS unit, most likely because both flow rates were considerably lower than the value calculated according to the *t_R_* criteria (270 g/min), as explained before (Table 1). Accordingly, the kinetic curves attained in the LS unit were considerably delayed in comparison with the SS unit (Figure 2). The CO_2_ flow rate of 85 g/min resulted in 85.3% oil recovery after 240 min of extraction (CO_2_/larvae ratio = 45.3 g/g), and a recovery of 90.9% was obtained with 130 g/min and 190 min (CO_2_/larvae ratio = 54.9 g/g). Thus, similar CO_2_/larvae ratios in the SS vessel and the LS vessel resulted in similar defatting degrees, although extraction time was doubled in the LS unit in comparison with the SS unit (Figure 2). 

Since 130 g/min was the maximum CO_2_ flow rate suitably controlled in the SFE unit, process conditions to study the defatting of the different *H. illucens* batches were set to 450 bar pressure, 60 °C temperature, 130 g/min CO_2_ flow rate, and 190 min extraction time. These conditions were kept constant to study the effect of oil and moisture content in the larvae supercritical defatting.

### 3.2. Extraction Yield and oil Recovery

Table 2 provides the results obtained after the supercritical CO_2_ defatting of eight batches of *H. illucens* larvae with different content of oil (16.80–29.17% w/w) and moisture (4.45–15.95% w/w). In addition, the extraction yield (Equation (1)), oil content in the defatted flour (% w/w), and percentage of defatting calculated according to Equations (2) and (3) are also given in Table 2. Taking into account the amounts of material recovered in the separators (S1 and S2) together with the mass of defatted flour recovered, the mass balance exhibited an accuracy larger than 94%.

**Figure 3:**
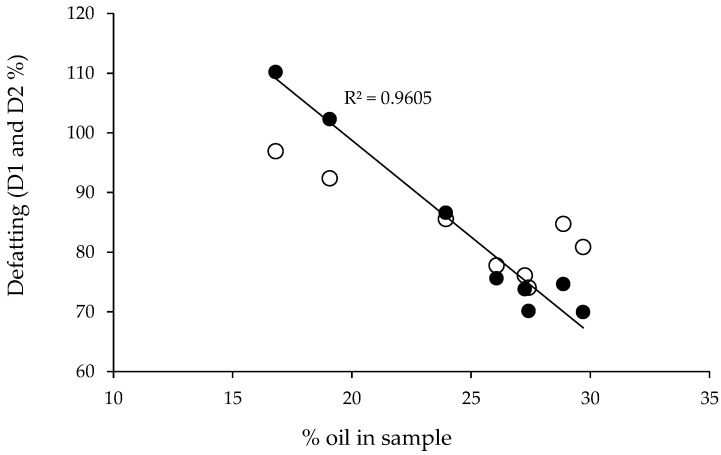
Supercritical defatting attained D1 (⬤) and D2 (◯) in different samples of *H. illucens* larvae as a function of the initial oil content (% w/w) of the flour. Solid line: D1 linear correlation. Extraction conditions (1350 cm^3^ vessel): 450 bar, 60 °C, 450 g larvae, 130 g/min CO_2_, and 190 min.

As a general trend, it can be stated that the defatting achieved (D1 and D2) increased according to the decrease in the oil content in the larvae sample, as depicted in Figure 3. Differences between D1 and D2 (which were lower than 13%) and D1 values larger than 100% probably occurred due to the coextraction of moisture and other minor CO_2_-soluble compounds present in the flour. Furthermore, D1 values were linearly correlated with the% w/w oil content, with a regression coefficient of R^2^ = 0.9605. 

Although a defatting ca. 90% was expected to be obtained according to the analyses presented in Section 3.1 using a *H. illucens* sample with high content of oil (47.3% w/w), not all the experiments carried out with samples 1–8 (Table 2) attained such a high defatting degree. Then, it may be supposed that the presence of moisture in the larvae batches might be a potential factor that influenced the supercritical CO_2_ defatting process.

**Figure 4:**
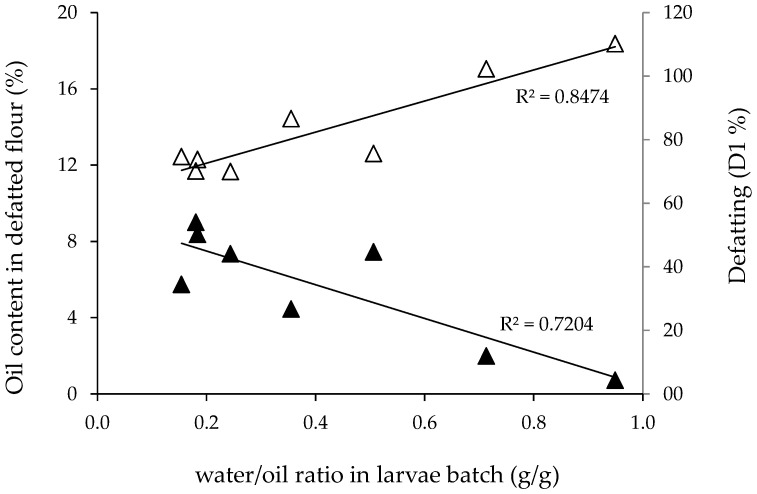
Oil content (% w/w) in the defatted flour (▲) and oil recovery (D1 values) (△) obtained from the different *H. illucens* larvae batches as a function of their moisture/oil ratio. Extraction conditions (1350 cm^3^ vessel): 450 bar, 60 °C, 450 g larvae, 130 g/min CO_2_, and 190 min. Solid lines: linear regressions.

Figure 4 shows the remaining concentration of oil in the defatted flour and the D1 values of the experiments as a function of the moisture/oil ratio (g/g) of the larvae batch. As a general tendency, increasing moisture/oil ratios resulted in higher D1 vales and consequently lower oil contents in the defatted flour. Then, it can be concluded that the presence of moisture in the larvae batch, in the range of values studied, did not negatively affect the efficacy of the oil extraction, which was mainly determined by the initial content of oil in the sample. 

Nevertheless, the degree of defatting produced in the *H. illucens* larvae with 47.3% w/w of oil could not be attained in any of the batches (1–8) of Table 2, which contained lower oil concentrations and similar or higher moisture (% w/w). In this respect, it is relevant to highlight that the larvae microstructure might also affect the supercritical defatting process because it affects the porosity of the packed bed. A lower apparent density resulted in higher packed bed porosity; thus, the efficacy of the supercritical extraction process was improved. Consequently, the apparent density of the different *H. illucens* larvae was measured by determining the weight of grounded larvae necessary to fill a 10 mL test tube (2 cm diameter). The apparent density of the larvae with high oil content (47.3% w/w) was 0.342 ± 0.014 g/cm^3^, while the apparent density of the batches 1–8 (Table 2), with 16.80–29.17% w/w oil content, were in the range of 0.411–0.479 g/cm^3^. Therefore, the *H. illucens* larvae with 47.3% w/w of oil and 0.342 ± 0.014 g/cm^3^ apparent density resulted in a higher defatting degree than that obtained from *H. illucens* batches with lower oil contents but higher apparent densities. That is, the differences in the microstructure of the packed beds may explain the difficulty of attaining 90% defatting in batches 1–8, and only those samples with low oil content were defatted up to 90% or even higher. 

### 3.3. Oil Composition, Moisture, and Peroxide Value

#### 3.3.1. Fatty Acid Profile 

Table 3 presents the fatty acid profile of the different oils obtained by supercritical CO_2_ extraction. Considering the percentage of saturated, monounsaturated, and polyunsaturated fatty acids (SFA, MUFA, and PUFA, respectively), no relevant variations in the fatty acid profile were observed. 

Therefore, regardless of the content of oil or moisture in the larvae batch processed, all the oils obtained presented the typical fatty acid composition described for *H. illucens*, which has a high content of lauric acid, this being the main fatty acid (c.a. 50% w/w), as well as palmitic acid, myristic acid, oleic acid, and linoleic acid.

#### 3.3.2. Minor Lipid Compounds

The content of two minor lipid compounds (squalene and phytosterols) present in the larvae batches and the extracted oils was determined by GC–MS. A slight trend toward a higher concentration of squalene and phytosterols was associated with increased values in the percentage moisture of the larvae batch. Nevertheless, an enhancement of phytosterols or squalene extraction due to the presence of moisture should not be expected, because moisture is not a good cosolvent for the supercritical CO_2_ extraction of these compounds [32].

**Figure 5:**
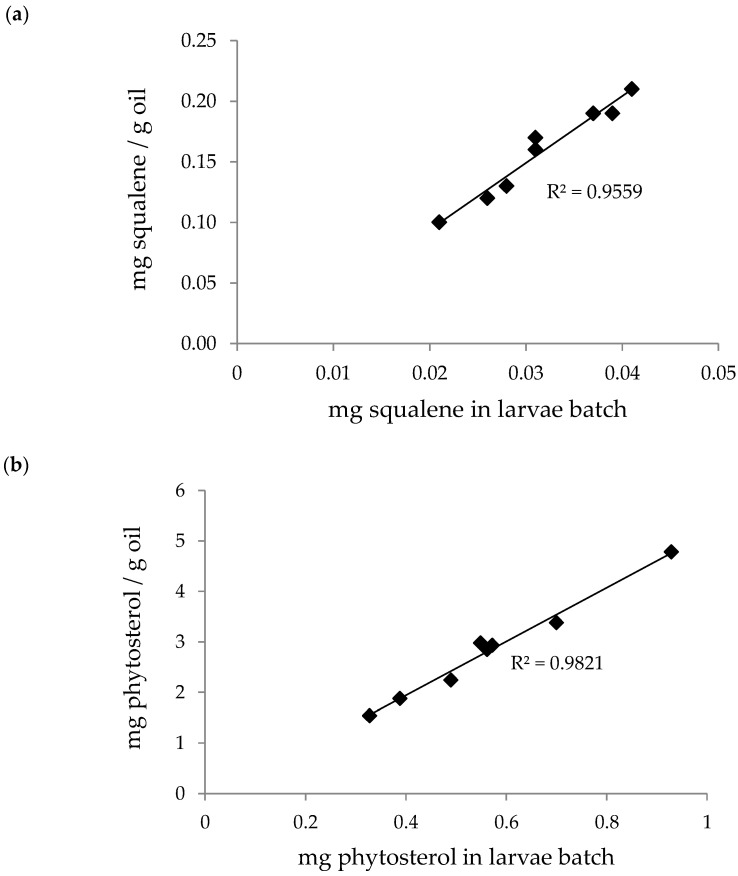
Squalene (**a**) and phytosterol (**b**) content (mg/g) in the extracted oils from the different *H. illucens* batches as a function of their content (mg) in the larvae sample. Extraction conditions (1350 cm^3^ vessel): 450 bar, 60 °C, 450 g larvae batch, 130 g/min CO_2_, and 190 min.

Figure 5 shows the concentration of squalene (Figure 5a) and phytosterols (Figure 5b) in the extracted oils as a function of the minor lipid compound content in the initial larvae batches. As expected, a higher content of squalene or phytosterols in the initial insect flour resulted in a higher concentration of the compound in the extracted oil. In particular, the content of squalene was not remarkable in comparison to other edible oils, e.g., olive oil, which contained amounts ranging from about 100 to 800 mg/100 g [33]. However, the phytosterol content can be highlighted, with values around 200–400 mg/100 g. It is well known that phytosterols are not typically found in animal fats, including insects. However, *H. illucens* is a particular edible insect with the ability to accumulate phytosterols, as reported previously by other authors [34,35], who described similar values to those discussed in this study. Comparing the obtained phytosterols values to those usually present in main vegetable oils, it was observed that *H. illucens* contents were higher than those in palm oil (~70–80 mg/100 g) or olive oil (~200 mg/100 g), and similar to those in soybean oil (~300 mg/100 g) or sunflower oil (~300–400 mg/100 g), as reported by Verleyen et al. [36]. Therefore, it can be concluded that it is possible to obtain a fat from *H. illucens* using SFE containing the typical minor lipids of interest at values that are proportional to the initial content in the larvae, regardless of the initial moisture and fat content.

#### 3.3.3. Moisture Content of Oils

As indicated above, the different larvae batches contained different initial moisture levels (Table 2); thus, the oil extracted could be expected to contain variable contents of moisture. The determination of the moisture content in the oils is relevant since lower moisture levels of the oil indicate higher quality.

**Figure 6:**
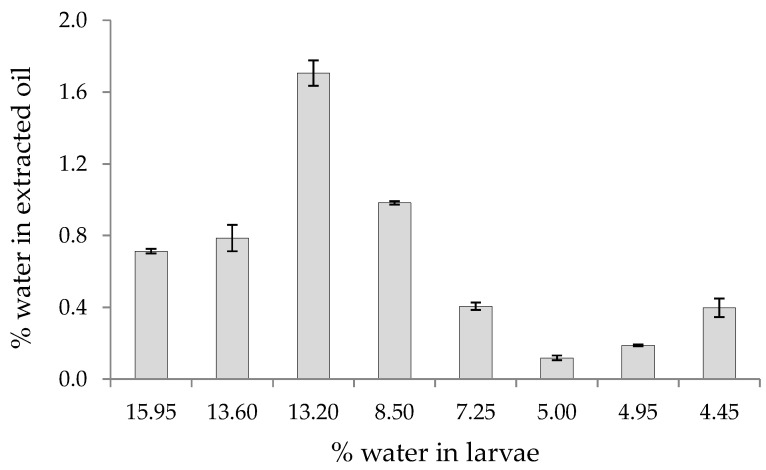
Moisture content (% w/w) in the oil extracted from the different samples of *H. illucens* larvae as a function of the initial moisture content in the larvae.

As shown in Table 2, variable moisture contents with relevant differences were determined in the oil samples. Additionally, as shown in Figure 6, although no linear tendency could be established, as a general trend, it can be stated that a higher content of moisture in the oil extracted was observed when higher amounts of moisture were present in the *H. illucens* larvae batch. 

Moisture values were in the range of 0.118–1.706% w/w. Currently, there is a lack of specific recommendations or standards for moisture content of oils from edible insects. Nevertheless, the Codex Alimentarius standard [37,38] for “edible fats and oils not covered by individual standards” recommends a matter volatile at 105 °C of 0.2%, which would include moisture. Taking this value as reference, only oil samples 5 and 6 would show a proper quality from the moisture point of view (Table 2). These samples were obtained from larvae that contained initial moisture values ≤ 5%. Therefore, it could be suggested that, in order to obtain oils from *H. illucens* with proper moisture quality using SFE as defatting procedure, an efficient drying of the initial larvae to reach values ≤ 5% seems to be necessary. Otherwise, for higher larvae moisture, a refining process of the SFE extracted oil could be required (e.g., oil blanching). The comparison with other methods to defat edible insects would be of interest at this point (e.g., mechanical pressing), in order to evaluate whether the observed result would be a specific limitation of the SFE, or if it is mainly related to the initial moisture content of the larvae, regardless of the defatting method. 

#### 3.3.4. Peroxide Index of Extracted Oils

**Figure 7:**
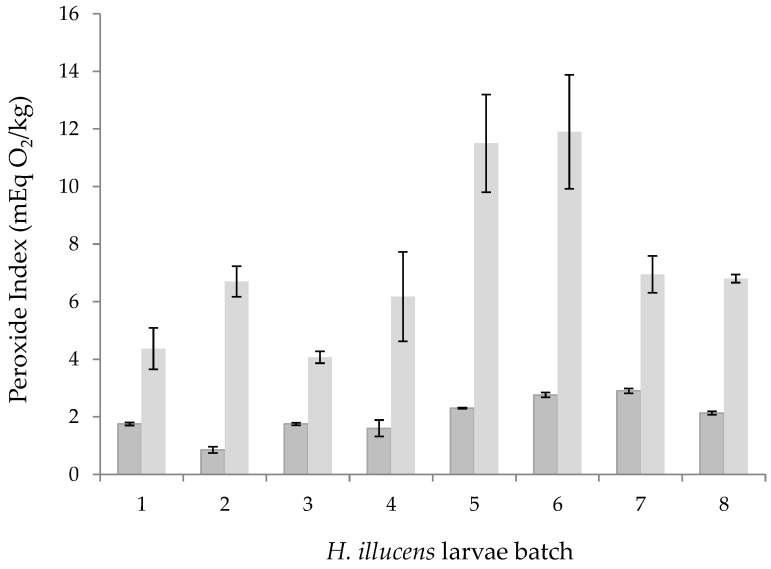
Peroxide Index of the oils extracted by SFE (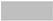) and the oils extracted using hexane (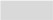).

Figure 7 shows a comparison of the peroxide index of the oils extracted by SFE and the oils extracted using hexane. The peroxide values of the supercritical oils were in the range 0.85–2.76 mEqO_2_/kg, which were considerably lower than the values obtained by extraction with hexane (4.07–11.90 mEq of O_2_/kg).

Although maximum peroxide levels for insect fats have not yet been specified and no regulations have been established in this regard, all the values of the samples from SFE were below 5 and 10 mEq O_2_/kg, which are the maximum values referred for fish oil and for edible fats not covered by specific quality standards, respectively [37,38]. On the contrary, samples from hexane defatting exceeded these references in most cases. Therefore, regardless of the initial fat and moisture content of larvae, the SFE allowed obtaining oils with a proper quality from the oxidative point of view, with the SFE being more appropriate than conventional defatting by hexane. 

## 4. Conclusions

Eight different batches of *H. illucens* larvae (16.80–29.17% w/w oil) were defatted with supercritical CO_2_ at 450 bar and 60 °C, according to the pressure and temperature conditions established in a previous work. The variable oil and moisture content in the samples resulted in insect flours with a variable degree of defatting (74–97%), obtaining increasing oil recoveries with decreasing values of the percentage of oil in the larvae. Since the presence of moisture may inhibit the supercritical extraction of oils, higher moisture content (lower oil content) in the larvae batch may result in lower defatting. Nevertheless, according to our study, the presence of moisture in the larvae batch, in the range of values studied (up to 16% w/w), did not interfere the efficacy of the supercritical oil recovery. Nevertheless, moisture content in the oils attained values up to 1.7% w/w, being not desirable from a quality point of view.

No differences were determined in the oil fatty acid profiles, which were all characterized by a high content of lauric acid (c.a. 50% w/w). The presence of squalene and phytosterols was also determined in the oils, and the concentration of these minor lipid compounds was mainly determined by their content in the larvae batch. Furthermore, the peroxide index of the oils extracted by supercritical CO_2_ was in the range of 0.85–2.76 mEq O_2_/kg, being much lower than the peroxide index of the oil obtained when the larvae were defatted using hexane.

## Figures and Tables

**Table 1:** SFE *H. illucens* scaling-up at 450 bar and 60 °C (CO_2_ density = 923 kg/m^3^).

	SS (270 cm^3^)	LS (1350 cm^3^) Calculated	LS (1350 cm^3^) Experimental
F = larvae mass (g)	100	450	450
D (cm)	4.3	6.7	6.7
L (cm)	18.8	38.3	38.3
A = vessel cross-flow area (cm^2^)	14.5	35.3	35.3
Q (g/min)	60.0	270.0	130.0
v (cm/min) = Q/(A·ρ)	4.48	8.29	4.00
t_R_ (min) = F/Q	1.67	1.67	3.46

**Table 2:** Supercritical CO_2_ defatting of different batches of *H. illucens* larvae at 450 bar, 60 °C, 450 g larvae, 130 g/min CO_2_, and 190 min (1350 cm^3^ vessel).

Sample	% Oil in Sample	% Moisture in Sample	Mass Recovered in S1 (g)	Mass Recovered in S2 (g)	Defatted Sample (g)	Yield (%)	Oil Recovery (D1%)	% Oil in Defatted Sample	Defatting (D2%)	% Moisture in Oil Recovered
1	16.80	15.95	83.3	12.8	326.6	18.5	110.2	0.71	96.9	0.713
2	19.07	13.60	87.8	15.9	330.0	19.5	102.3	1.98	92.4	0.786
3	23.94	8.50	93.3	4.1	349.4	20.7	86.6	4.45	85.6	0.983
4	26.07	13.20	88.7	4.8	350.0	19.7	75.6	7.45	77.8	1.706
5	27.41	4.95	86.5	2.5	355.5	19.2	70.1	9.00	74.1	0.188
6	27.25	5.00	90.5	2.0	350.2	20.1	73.8	8.37	76.1	0.118
7	28.87	4.45	97.0	2.9	345.4	21.6	74.7	5.74	84.7	0.397
8	29.07	7.25	93.5	2.7	348.2	20.8	70.0	7.35	80.9	0.406

**Table 3:** Fatty acid profile and total content of saturated fatty acids (SFA), monounsaturated fatty acids (MUFA) and polyunsaturated fatty acids (PUFA) (% w/w) in the oils extracted from the different *H. illucens* larvae batches.

Sample	1	2	3	4	5	6	7	8
C10:0	0.7	0.8	1.1	1.0	1.2	1.1	0.9	0.9
C12:0	49.8	48.7	51.0	50.3	51.5	51.5	49.5	50.3
C14:0	10.8	10.5	10.1	10.1	10.0	9.9	10.0	10.2
C14:1	0.4	0.3	0.4	0.4	0.4	0.4	0.4	0.4
C16:0	14.7	14.4	12.8	13.0	12.8	12.7	13.5	13.2
C16:1	2.3	2.3	2.4	2.4	2.3	2.4	2.5	2.4
C17:0	0.4	0.5	0.4	0.4	0.4	0.4	0.4	0.4
C17:1	0.3	0.3	0.2	0.3	0.2	0.4	0.3	0.3
C18:0	2.8	2.7	2.2	2.3	2.3	2.3	2.4	2.3
C18:1	8.7	9.1	8.5	8.6	8.3	8.2	8.9	8.7
C18:2	5.8	6.6	7.3	7.5	7.3	7.2	7.6	7.4
C18:3 n-6	0.1	0.2	0.1	0.1	0.1	0.2	0.1	0.1
C18:3 n-3	2.9	3.2	3.3	3.4	3.2	3.2	3.3	3.2
C20:1	0.3	0.3	0.2	0.2	0.1	0.2	0.1	0.1
Total SFA	79.2	77.6	77.6	77.1	78.1	77.8	76.7	77.3
Total MUFA	12.0	12.4	11.7	11.9	11.3	11.6	12.2	11.9
Total PUFA	8.9	10.1	10.8	11.1	10.6	10.6	11.0	10.7

## Data Availability

The data are available from the corresponding author.
